# The Need for SMN-Independent Treatments of Spinal Muscular Atrophy (SMA) to Complement SMN-Enhancing Drugs

**DOI:** 10.3389/fneur.2020.00045

**Published:** 2020-02-03

**Authors:** Niko Hensel, Sabrina Kubinski, Peter Claus

**Affiliations:** ^1^Institute of Neuroanatomy and Cell Biology, Hannover Medical School, Hannover, Germany; ^2^Center of Systems Neuroscience (ZSN), Hannover, Germany

**Keywords:** spinal muscular atrophy, therapy, survival of motoneuron (SMN), network biology, systems biology, SMN-irreversibility, SMN-independency, neurodegeneration

## Abstract

Spinal Muscular Atrophy (SMA) is monogenic motoneuron disease caused by low levels of the Survival of Motoneuron protein (SMN). Recently, two different drugs were approved for the treatment of the disease. The antisense oligonucleotide Nusinersen/Spinraza® and the gene replacement therapy Onasemnogene Abeparvovec/Zolgensma® both enhance SMN levels. These treatments result in impressive benefits for the patients. However, there is a significant number of non-responders and an intervention delay has a strong negative impact on the efficacy. Obviously, later stages of motoneuron degeneration cannot be reversed by SMN-restoration. Therefore, complementary, SMN-independent strategies are needed which are able to address such SMN-irreversible degenerative processes. Those are defined as pathological alterations which are not reversed by SMN-restoration for a given dose and intervention delay. It is crucial to tailor SMN-independent approaches to the novel clinical situation with SMN-restoring treatments. On the molecular level, such SMN-irreversible changes become manifest in altered signaling modules as described by molecular systems biology. Based on our current knowledge about altered signaling, we introduce a network approach for an informed decision for the most potent SMN-independent treatment targets. Finally, we present recommendations for the identification of novel treatments which can be combined with SMN-restoring drugs.

## Introduction

Spinal Muscular Atrophy (SMA) is a monogenic, autosomal recessive neurodegenerative disease. It has an incidence of 1:6,000−1:10,000, preferentially affects infants and is the most common rare disease in this age cohort (1, 2). The second motoneurons in the spinal cord and brain stem degenerate in SMA patients, resulting in fatigue, paralysis, and atrophy of the proximal muscles (3, 4). Patients harbor homozygous deletions or mutations of the *Survival of Motoneuron1* (*SMN1*) gene. The lack of the corresponding SMN protein causes SMA (5). However, all humans comprise a second very similar gene, *SMN2*, which encodes the same SMN protein. *SMN2* differs from *SMN1* in some mutations with a translational silent cytosine to thymine transition within exon 7 (6). This leads to an altered splicing of the vast majority of the *SMN2* pre-mRNA resulting in a shortened transcript which lacks exon 7 (Δ7 mRNA) and only a few transcripts of the mature full length *SMN2* mRNA (7). While the full-length protein is stable, the SMNΔ7 protein is rapidly degraded. As a consequence, *SMN2* produces only about 10–15% of the protein amount compared to *SMN1*. The *SMN2* gene is not able to fully compensate the *SMN1* loss in patients leading to the preferential degeneration of motoneurons (6, 7).

The number of the *SMN2* gene copies is the most potent genetic modifier of SMA severity (8): the number of gene copies negatively correlates with disease severity. SMA is divided into five different subtypes based on the clinical picture and defined by the age of disease onset, the life expectancy and the motor function milestones which the patients are able to reach (9–11). The most severely affected SMA type 0 patients decease before or within the first month after birth (12). The most common subtype is the severe SMA type 1 with symptoms occurring within the first 3 months after birth. These Patients never gain the ability to sit or to control their head and die within the first 2 to 3 years of life (11, 13). Intermediate type 2 patients show the first symptoms in early childhood between the sixth and eighteenth months of age, are never able to stand and suffer from a marked reduction of life-expectancy (10). Symptoms in mild subtype 3, the juvenile form, typically occur after 18 months of age and these patients can stand and walk independently (10, 11, 14). In contrast, type 4 patients show mild muscle weakness symptoms in adulthood (10).

SMN is a multifunctional protein which localizes to the nucleus, cytoplasm, axon, and the neuromuscular junction (15–18). The loss of more than one of these functions likely contributes to motoneuron degeneration. The multifunctionality of SMN has been excellently reviewed elsewhere (19). Here, we exemplify two different functions: SMN is part of the machinery which assembles spliceosomal components (20). It has been hypothesized that this leads to a general splice deficiency and that motoneurons are specifically sensitive to that (21). However, splicing is not a process which is restricted to neurons or motoneurons. It may thus be possible that this is the “housekeeping” function of the SMN protein affecting all cells and organs. A more specific function is the involvement of the SMN protein in the neuronal actin cytoskeleton (22). SMN directly interacts with profilin2a, an actin-binding protein which is specifically expressed in neurons (23–25). A lack of the SMN protein leads to enhanced accessibility of profilin2a for its upstream Rho-kinase (ROCK). As a consequence, profilin2a becomes hyper-phosphorylated inducing a neuron-specific dysregulation of the actin cytoskeleton (22, 25–27).

The exact molecular mechanism of motoneuron degeneration remains elusive. However, pathohistology reveals distinct degenerative phenotypes in SMA patients which allow reconstructing a “natural history” of motoneuron degeneration (28). The pathohistology of SMA type 1 patients' spinal cords reveals two prominent characteristics of neurodegeneration: a loss of motoneurons in the anterior horn and a chromatolysis of some of the remaining motoneurons (29). The latter is a distinct degenerative process characterized by the loss of rough endoplasmic reticulum and a displacement of the nucleus toward the cell body periphery (30). Chromatolysis in SMA hints for a distal pathology with an axonal damage—an axotomy is the most simple method to experimentally induce chromatolysis in motoneurons (30). In such models, chromatolysis occurs before regeneration and degeneration. Thus, it is supposed that chromatolytic neurons are on the verge of cell death but that a regenerative potential remains. However, it is unclear whether chromatolytic motoneurons can be rescued or not in SMA. Evidence for an axonal dying back mechanism came from studies in fetuses predicted to develop SMA. Muscle histology revealed altered neuromuscular junction phenotypes (31). Moreover, central synapses were altered in pre-symptomatic SMA mice indicating a synaptic pathology (32). Thus, there may be a functional motoneuron degeneration preceding the loss of motoneurons which has been observed in *post mortem* pathohistology. However, such studies indicated even earlier perturbations in motoneuron development: SMA type 1 and 2 patients displayed heterotopic motoneurons with a round-shaped migratory phenotype and an abnormal localization at the anterior rim of the spinal cord (28).

The SMN protein is ubiquitously expressed and it is not surprising that a lack of SMN protein affects peripheral organs in SMA patients. Those include metabolic alterations (33), muscle (34), heart (35), vasculature (36, 37), pancreas (38), and liver (39). Accordingly, SMA is considered to be a multi-system disease (40, 41). However, motor impairments and muscle atrophy are severe conditions in patients. It is difficult to dissect peripheral organ-intrinsic pathomechanisms from the neuromuscular phenotype. In experimental SMA models, such as SMA mice, it is possible to perform an organ or cell-specific rescue approach which selectively restores SMN protein levels in single cell types. Thereby, some studies showed cell- or organ-intrinsic pathomechanisms including muscle (42, 43) and astrocytes (44). Considering the relation between SMN levels and peripheral organs it has been suggested that there are cell or organ-specific SMN thresholds needed for proper organ function (45). However, motoneurons are preferentially affected independent of the clinical type and are thus an important and common therapeutic target. An elevation of SMN levels in the spinal cord has been a successful strategy for novel therapeutics approved recently.

In this state-of-the-art review, we focus on the approved compounds Nusinersen/Spinraza® and Onasemnogene Abeparvovec/Zolgensma® which both enhance SMN levels in the central nervous system (CNS). Thereby, we exemplify that (i) *central SMN-restoration* in the CNS is important but may not be sufficient because (ii) a *peripheral SMN-restoration* may be needed in patients with very low peripheral SMN levels. Moreover, (iii) a *SMN-independent regeneration* has to be considered in patients with a delayed therapeutic intervention. We will then focus on such SMN-independent approaches. Moreover, we review pre-clinical studies which used interventions in cellular signaling as a strategy to identify SMN-independent treatment options. Thereby, we include pathways with evidence from mammalian SMA models only. Finally, we summarize the experience with SMN-independent pre-clinical approaches with a set of recommendations for their future identification.

## Current Therapies Focus on SMN Levels in the CNS: The Importance of Peripheral SMN-Restoration and SMN-Independent Regeneration for Future Therapies

Since the identification of the causality between SMN protein loss and SMA, many treatment strategies focused on restoring SMN levels. Those strategies are generally termed “SMN-dependent.” Since motoneurons are preferentially affected independent of the clinical subtype, they are a common target for a SMN-restoration in the central nervous system and this strategy is important for all SMA cases irrespective of the severity (Table 1). In December 2016, the first treatment for all clinical subtypes of SMA was approved by the US Food and Drug Administration (FDA) and half a year later for Europe as well. The drug Nusinersen/Spinraza® is an antisense oligonucleotide (ASO) which does not cross the blood-brain-barrier. The ASO is directly administered to the CNS by lumbar puncture into the cerebrospinal fluid (CSF) where it enhances central SMN levels only (46). On the molecular level, Nusinersen/Spinraza® modulates the deficient *SMN2* pre-mRNA splicing to restore SMN protein levels (47). The half-life time of the ASO within the spinal cord of non-human primates is about 140 days allowing a reduction of application burden by lumbar punctures (48). Currently, Nusinersen/Spinraza® is administered to SMA patients by four loading doses followed by maintenance doses every 4 months (49). In a phase-I study, Nusinersen/Spinraza® enhanced the survival compared to natural history data. The ASO was detected in neurons and glia cells of deceased SMA patients' spinal cords where it induced exon 7 inclusion (46). However, low ASO-levels could also be found in blood indicating some leakiness during the ASO administration procedure or a subsequent CSF clearance into the blood (49, 50). Although there was a significant number of non-responders during the observation period of a placebo-controlled study, over 70% of treated infants improved motor functions and the risk of death or permanent assisted ventilation dropped by 47% compared to the placebo group (49).

**Table 1:**
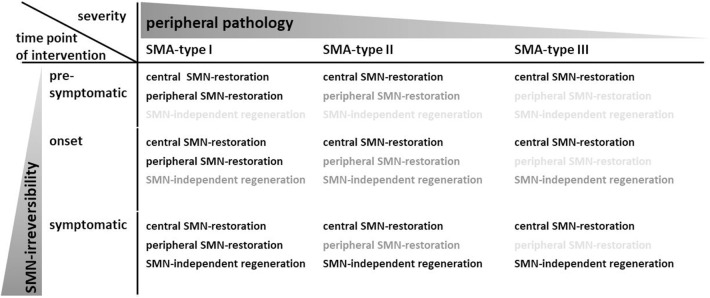
Severity and time point of intervention critically determines the treatment strategy.

*Central SMN-restoration in the CNS is needed in all patients (black), since SMN reduction commonly affects motoneurons in the spinal cord. According to the hypothesis of SMN organ-specific thresholds, more severely affected patients with a strong SMN reduction may be more susceptible for peripheral organ defects. While this makes a peripheral SMN-restoration important in SMA type I patients (black), this is less important for SMA type II patients (dark gray) and possibly neglectable for milder affected patients (light gray). Moreover, later disease stages show pathological changes which cannot be reversed by SMN-restoration only—they are SMN-irreversible. As a consequence, SMN-independent strategies are needed which allow reversal of symptoms or regeneration. While pre-symptomatically treated patients may not depend on such strategies (light gray), this may be more important for patients which are treated at disease onset (dark gray), and critical for symptomatically treated patients (black). In summary, different patient cohorts with different needs for treatments can be defined. It is likely that a single compound may not address central and peripheral SMN-restoration at the same time. Moreover, a combinatorial treatment regimen should include regenerative SMN-independent drugs*.

In 2019, the FDA approved Onasemnogene Abeparvovec/Zolgensma®, an Adeno-associated virus 9 (AAV9) delivering a cDNA which codes for the SMN protein, as a gene replacement therapy. Onasemnogene Abeparvovec/Zolgensma® is systemically applied to children less than 2 years. So far, there is one published phase-I study with the AAV9 employed at two different doses which resulted in improvements in motor function and survival compared to natural history data (51). The AAV9 crosses the blood-brain barrier which induces SMN expression in the CNS and in peripheral organs. However, human bioavailability data have not been published so far. Moreover, AAV9 does not integrate into the genome which leads to a dilution effect in mitotic cells. As a consequence, a SMN-restoration by a gene replacement therapy may be of limited sustainability in the periphery. However, in the light of the multi-system character of SMA, a *peripheral SMN-restoration* may be needed complementing the central SMN-restoration in the CNS (Table 1). According to the hypothesis of organ-specific SMN thresholds, severely affected children with very low peripheral SMN levels are potentially at risk of multi-organ defects while this could be less relevant in milder affected patients.

**Figure 1:**
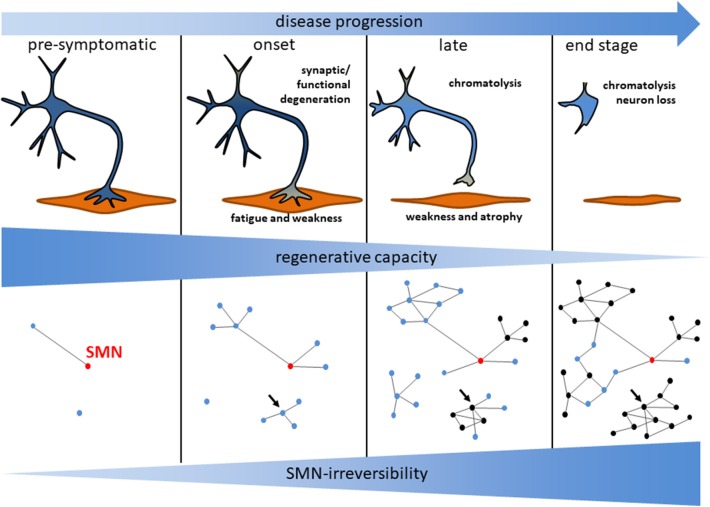
Model for motoneuron degeneration and the underlying signaling network in SMA. Motoneuron loss observed in *post mortem* analyses is preceded by a functional degeneration of central synapses and the neuromuscular junction. The subsequent axonal damage induces a chromatolytic phenotype of the motoneurons. During disease progression those processes become less reversible indicated by a reduced regenerative capacity. This is reflected by a growing network of dysregulated signaling nodes with an increased fraction of SMN-irreversible (black) vs. SMN-reversible (blue) signaling mediators. SMN-restoration restores blue nodes only. The relative number of SMN-restorable nodes becomes reduced over time as illustrated in this hypothetical scheme. Highly connected SMN-irreversible (black) nodes may be potent treatment targets (arrow). Those nodes may be critical regulators for a module involved in a specific degenerative process.

Importantly, the beneficial effects of Nusinersen/Spinraza® are dependent on disease duration at the time of intervention: the shorter the infants were symptomatic before treatment; the higher was the survival without permanent ventilation as well as the improvement of motor functions (49, 52). This effect was strongest in pre-symptomatic patients with two or three *SMN2* copies resulting in impressive benefits and the achievement of motor milestones such as independent walking (53). Similarly, patients treated early with Onasemnogene Abeparvovec/Zolgensma® performed better compared to patients with delayed intervention (51). Taken together, this confirms a number of pre-clinical studies which employed SMN-dependent treatment strategies in SMA mice: disease duration before treatment is critical and a delayed intervention leads to a less efficient rescue (54). This and the occurrence of non-responders clearly demonstrate pathological processes which cannot be reversed by enhancing SMN protein levels and that they increase in number and/or severity with prolonged intervention delay (Figure 1). It is unclear which pathological changes underlie this *SMN-irreversibility*. However, the complete loss of a motoneuron is a clearly irreversible change. Moreover, this constitutes a severe problem if no newborn screening becomes implemented and for milder affected SMA patients which are symptomatic for years. Complementary strategies are needed which are not based on enhanced SMN protein expression—since this is already accomplished by Nusinersen/Spinraza® or Onasemnogene Abeparvovec/Zolgensma®—but reverse pathological changes *independent of SMN* (Table 1).

## An “SMN-Independent Treatment Approach” Does not Necessarily Mean That it is Independent From SMN Levels: The Importance of SMN-Irreversible Processes

Since the introduction of SMN-enhancing drugs with an impressive but yet limited effect, SMN-independent treatment approaches attained more attention. Those could be combined with SMN-enhancing drugs for defined patient cohorts (Table 1). However, the definition of an “SMN-independent treatment approach” is not clear yet. Sometimes, a strategy which does *not* involve the increase of the SMN protein level is included in such definition. This is misleading because there might be strategies which do not rely on enhanced SMN levels but are still SMN-dependent. For example, inhibition of Rho-kinase (ROCK) activity (see below) could be considered as an SMN-independent treatment strategy. However, its activity toward different downstream targets has been demonstrated to be SMN-dependent by direct interaction of SMN with profilin2a, a ROCK binding protein (25). Neurodegeneration could elicit pathway and network perturbations which could not be restored by therapeutic SMN-enhancing intervention (Figure 1). Those signaling modules represent important putative targets for combinatorial treatments. For an optimal outcome, treatment strategies should be adapted to specific pathological changes of a disease. In principle, it is possible that SMN restoration induces a regenerative process which abrogates those changes: the pathological process or symptom may be reversed. Thus, SMN-independent strategies should focus on treatment targets which are *SMN-irreversible* (Figure 1). A pathomechanistic SMN-reversible change may have already changed back to normal levels by application of antisense oligonucleotide or gene replacement therapies so that there is no process to reverse anymore. This also applies to strategies which target peripheral tissues without enhancing SMN levels. Pre-clinical studies in SMA mice specifically target muscle function with myostatin inhibitors enhancing body- and muscle weight. However, the clinically relevant impact on motor functions is unclear yet (55, 56). Moreover, SMN-irreversibility of the underlying pathological changes such as the loss of muscle function has not been reported so far: It is possible that a central and peripheral restoration of the SMN levels may enhance the muscle functions in a way that myostatin inhibition would not have an extra benefit. As seen by the better response of early treated patients, SMN-irreversibility critically depends on the intervention delay. Thus, an appropriate SMN-independent treatment strategy must rely on a given intervention delay (Figure 1): In fact, a pre-clinical pipeline for the development of combinatorial treatment approaches should detect alterations *downstream* of SMN deficiency to identify a specific pathomechanistic change (a target), test its *SMN-irreversibility* in a model reflecting the clinical situation with delayed intervention, and show *robust pathophysiological benefits* when rescued. In the last years, we and others identified several signaling pathways which are potential treatment targets for Spinal Muscular Atrophy without changing SMN levels. However, there are only limited reports about their SMN-irreversibility.

## Intervention in Cellular Signaling as a Strategy for SMN-Independent Treatment Approaches: A Skeptical View on our Current Knowledge About Pathways

There are a number of potential SMA treatment approaches which rely on their interference with cellular signaling. Thereby, two different strategies have been followed: approaches which interfere with an *altered* pathway downstream of SMN-deficiency and potential treatments focusing on unchanged pathways in SMA. The latter are more likely to be unaffected by SMN-restoration but may be less efficient. However, SMN-irreversibility of these approaches has not been tested yet and this is a pre-requisite for their SMN-independency. Those include the Rho kinase (ROCK) (25, 62–65), the extracellular regulated kinase (ERK) (62, 63, 66, 67), the c-Jun N-terminal Kinase (JNK) (68, 69), and the p53-pathway (70) (Box 1). The Phosphatase and tensin homolog (PTEN) pathway was not altered in SMA but its inhibition may exert some beneficial effects (71). PTEN is a pro-apoptotic protein, involved in Akt signaling. In an ischemic rat model, the death of hippocampal neurons was inhibited by decreased PTEN activity demonstrating general neuroprotective properties of PTEN inhibition (72). However, there are no findings of an altered PTEN signaling in SMA mice. A systemic AAV-based knock-down strategy led to a modest effect on survival and motor functions in SMNΔ7 mice (71). However, it is unclear whether motoneurons mediate those beneficial effects. Moreover, a systemic approach is based on high viral loads and may enhance the risk for cancer especially in non-neuronal cells.

Box 1 **An informed decision for the most promising candidate—the advantages of network biology.**

Network biology describes the physical and/or functional interaction of a plethora of biological molecular entities. An important application is the network biology of proteins involved in signaling (57). Signaling proteins form nodes which are connected by links or edges. Those edges define the relationship between the nodes. Those can be functional or physical interactions of proteins, normally derived from databases such as the BioGRID, Intact or EMBL-STRING databases (58–60). Graphical analysis algorithms are able to arrange the nodes based on their connectivity. This leads to the formation of modules or clusters which often share a common biological function (57). Hubs are highly connected nodes located within modules—thus being critical master regulators for distinct biological processes. Inter-modular nodes may even be more important since they affect more than one module simultaneously. If applied on a disease, network biology provides a non-reductionist view on molecular processes such as altered signaling. The selection of hubs or inter-modular nodes allows an informed decision for a novel treatment target.

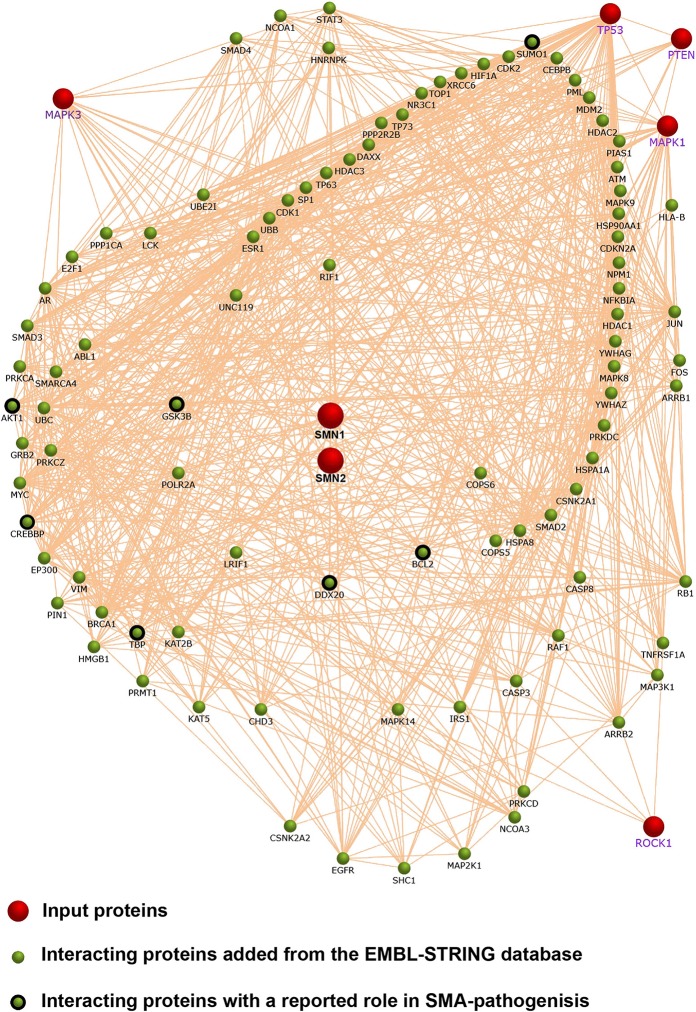

We performed a network analysis based on published reports about altered signaling in SMA. Therefore, we included SMN, JNK3 (MAPK3), p53 (TP53), PTEN, ERK (MAPK1), and ROCK1 as input proteins (red dots). We used the Functional Enrichment Analysis Tool (FunRich) (61) with the EMBL-STRING database for a network analysis (60). Highly connected interactors were added to the network (green dots). Interestingly, a number of them have been connected to SMA pathology before (black outlined green dots). However, the selection of the input proteins may be biased *a priori*. It is not always clear whether those pathways have been identified in hypothesis-free approaches. An unbiased high-throughput approach may circumvent this drawback.

Another possible target for a SMN-independent treatment is the c-Jun N-terminal kinase 3 (JNK3). JNK3 is a MAP-kinase which is specifically expressed in the central nervous system (CNS) where it becomes up-regulated after traumatic brain injury (73, 74). Within the CNS, it may be expressed in neurons, oligodendrocytes, and astrocytes (75–77). JNK inhibition in an Alzheimer's disease (AD) model partially restored synaptic dysfunctions (78) which are of particular interest with regard to the SMA pathology. As mentioned above, synaptic dysfunctions are an important hallmark of motoneuron degeneration in SMA. Interestingly, SMNΔ7 mice displayed an enhanced JNK3 activation in the spinal cord with an unknown cell type origin. However, enhanced JNK3 activity was measured in severely symptomatic post-natal day 12 mice indicating a reaction to a neurodegenerative trauma rather than being the cause of motoneuron degeneration (68, 69). Altered JNK phosphorylation levels could not be detected in SMA cellular and mouse models or human tissue (79). However, there might be effects in different segments of the spinal cord with the lumbar part showing highest susceptibility. For example, such a pattern has been observed for p-ERK in lumbar segments L3 and L5 (but not in thoracic or L1 segments) (63). Importantly, SMNΔ7 mice with a JNK3 knock-out lived significantly longer than control SMNΔ7 mice (68) while a pharmacological approach with a pan JNK inhibitor did not convincingly enhance their survival (69).

p53 is a major regulator of cell cycle, DNA repair and apoptosis in numerous cell types. In differentiated neurons, p53 may regulate neuronal regeneration vs. apoptosis which is promoted by p53 phosphorylation of N-terminal residues (80). *Post mortem* analysis of SMA patient spinal cords revealed a nuclear accumulation of p53 in motoneurons (81). This was corroborated in SMNΔ7 mice in which nuclear accumulation was accompanied by a specific phosphorylation at the N-terminus (70). Interestingly, the SMN protein directly interacts with p53 (82). However, this interaction has not been linked to the altered N-terminal p53 phosphorylation. Moreover, *in vivo* changes in p53 localization and phosphorylation were evaluated in symptomatic SMNΔ7 animals (70). Therefore, altered p53 homeostasis may be the result rather than the cause of motoneuron degeneration. Indeed, amino-terminal p53 phosphorylation is a cell-type specific signal for apoptosis (83) and may become activated due to the degeneration of motoneurons in later stages. In line with that, p53 inhibition rescued motoneuron numbers but not the motor functions and SMNΔ7 mice did not survived longer (70).

One of the first possible SMN-independent targets characterized was the RhoA kinase (ROCK) pathway. ROCK is an important regulator of neuronal actin dynamics which is critical for the function of motoneuron synapses (22). The level of ROCK activity was enhanced in the spinal cord of the intermediate *Smn*^2*B*/−^ SMA-mouse model at pre-symptomatic time points (64) but at no time point in the severe Taiwanese mice neither in spinal cord nor muscle (our data, not shown). Consistently, *Smn*^2*B*/−^ mice displayed elevated survival rates when treated with small molecule ROCK inhibitors (64, 65) while there were detrimental effects on severe Taiwanese mice (63). ROCK-inhibited *Smn*^2*B*/−^ mice did not show elevated SMN levels compared to control *Smn*^2*B*/−^ mice. However, SMN-irreversibility has not been checked for the enhanced ROCK activity and the SMN-independency of this effect is therefore not clear yet (64). The changes in ROCK activity have been mechanistically linked to a direct interaction of the SMN protein with the ROCK-target profilin2a: a lack of the SMN protein results in an enhanced binding of ROCK to profilin2a which subsequently mediates the pathological changes (25). Given this molecular model, a restoration of the SMN-levels would also restore dysregulated actin dynamics—this mechanism and the resulting treatment approach would not be SMN-independent. However, the cellular source of the enhanced ROCK activity has not been identified yet. Neurodegeneration often induces a chronic, detrimental neuroinflammation driven by an enhanced glial ROCK activity (84). Enhanced ROCK activity in intermediate *Smn*^2*B*/−^ mice may thus reflect neuroinflammation while it is possible that severe Taiwanese SMA mice die before developing such a chronic condition. Chronic neuroinflammation is a process which—once induced by neurodegeneration—sustains itself by inducing further neurodegeneration. This process may not be SMN-irreversible thus being a candidate for an SMN-independent treatment approach.

ERK is another pathway which is up-regulated in SMN knock-down cells (66) and in the spinal cord of two different severe SMA mouse models (63, 67). The ERK pathway is a classical neurotrophic signaling pathway and a positive regulator of neuronal regeneration (85). However, dependent on the localization of activated ERK, it may also trigger neurodegeneration (63). Inhibition of ERK enhanced survival of severe SMA mice and enhanced SMN expression accompanied by neuroprotection of motoneurons within the spinal cord (67). However, we employed a randomized study-design avoiding a litter-wise treatment and found a significant reduction in survival of severe Taiwanese SMA-mice treated with an ERK inhibitor (63). Thus, in our hands, we could indeed detect an enhanced ERK activity. However, ROCK as well as ERK inhibition was detrimental for SMA mice (63). ROCK and ERK are generic kinases expressed in most cells and tissues. In combination with a systemic inhibitory approach, this may produce significant side effects in a chronic treatment regimen. To develop a specific treatment strategy it is indispensable to identify and target the affected cell type in which the altered signaling event localizes. Moreover, ROCK and ERK act in a regulatory network influencing SMA-like pathophysiology in a combined manner rather than acting alone (63). Although both inhibitory regimens were detrimental, a *combination* of a ROCK and an ERK inhibitor performed better than the ERK inhibitor alone. This was accompanied by a ROCK-mediated rescue of the ERK activity in the spinal cord and confirmed *in vitro* studies in which we showed a crosstalk between both pathways (62, 63). This is a proof-of-principle of the network character of signaling events in SMA (Box 1).

## Outlook: A Set of Recommendations to Identify Novel Targets for Combinatorial SMA Treatment Strategies

Based on the previous comments, one can derive recommendations for the identification of novel signaling pathways as SMN-independent treatment targets:

- It is better to choose a SMA-specific alteration downstream of SMN-deficiency as a target rather than selecting an unspecific neuroprotective signaling pathway. It is more efficient to target SMA-specific pathological processes. Those translate into changed network modules.
- The selection of hubs or inter-modular nodes is a promising strategy for the identification of potent treatment targets. Targeting hubs or inter-modular nodes maximizes the beneficial effects on the whole network by restoring the equilibrium (Box 1).
- For a targeted approach excluding compensatory events or epiphenomena, a pre-symptomatic evaluation of the signaling is superior over symptomatic analyses.
- The signaling event must be localized in a disease relevant tissue and cell type. Motoneurons in the spinal cord and brain stem are still the preferred target in SMA. The identification of the cell type is most relevant for the pre-clinical experimental paradigm, since it allows a specific rescue approach thereby reducing side effects.
- The target must be SMN-irreversible which has to be tested in an appropriate model in combination with a SMN-enhancing drug. For the latter, suboptimal dosing or intervention delays have to be considered.
- Potency must be tested by a robust pathophysiological benefit in combination with SMN-enhancing drugs.

## Author Contributions

NH, SK, and PC wrote the manuscript. All authors contributed to manuscript revision, read, and approved the submitted version.

### Conflict of Interest

The authors declare that the research was conducted in the absence of any commercial or financial relationships that could be construed as a potential conflict of interest.

## References

[1] Prior TW. Spinal muscular atrophy: newborn and carrier screening. Obstet Gynecol Clin North Am. (2010) 37:23–36. 10.1016/j.ogc.2010.03.001 20494255

[2] Sugarman EA Nagan N Zhu H Akmaev VR Zhou Z Rohlfs EM. Pan-ethnic carrier screening and prenatal diagnosis for spinal muscular atrophy: clinical laboratory analysis of >72,400 specimens. Eur J Hum Genet. (2012) 20:27–32. 10.1038/ejhg.2011.134 21811307 PMC3234503

[3] D'amico A Mercuri E Tiziano FD Bertini E. Spinal muscular atrophy. Orphanet J Rare Dis. (2011) 6:71. 10.1186/1750-1172-6-71 22047105 PMC3231874

[4] Monani UR. Spinal muscular atrophy: a deficiency in a ubiquitous protein; a motor neuron-specific disease. Neuron. (2005) 48:885–96. 10.1016/j.neuron.2005.12.001 16364894

[5] Lefebvre S Burglen L Reboullet S Clermont O Burlet P Viollet L. Identification and characterization of a spinal muscular atrophy-determining gene. Cell. (1995) 80:155–65. 10.1016/0092-8674(95)90460-3 7813012

[6] Lorson CL Hahnen E Androphy EJ Wirth B. A single nucleotide in the SMN gene regulates splicing and is responsible for spinal muscular atrophy. Proc Natl Acad Sci USA. (1999) 96:6307–11. 10.1073/pnas.96.11.6307 10339583 PMC26877

[7] Vitte J Fassier C Tiziano FD Dalard C Soave S Roblot N. Refined characterization of the expression and stability of the SMN gene products. Am J Pathol. (2007) 171:1269–80. 10.2353/ajpath.2007.070399 17717146 PMC1988876

[8] Campbell L Potter A Ignatius J Dubowitz V Davies K. Genomic variation and gene conversion in spinal muscular atrophy: implications for disease process and clinical phenotype. Am J Hum Genet. (1997) 61:40–50. 10.1086/513886 9245983 PMC1715870

[9] Munsat TL Davies KE. International SMA consortium meeting. (26-28 June 1992, Bonn, Germany). Neuromuscul Disord. (1992) 2:423–8. 10.1016/S0960-8966(06)80015-5 1300191

[10] Finkel R Bertini E Muntoni F Mercuri E Group ESWS. 209th ENMC International Workshop: outcome measures and clinical trial readiness in spinal muscular Atrophy 7-9 November 2014, Heemskerk, The Netherlands. Neuromuscul Disord. (2015) 25:593–602. 10.1016/j.nmd.2015.04.009 26045156

[11] Wang CH Finkel RS Bertini ES Schroth M Simonds A Wong B. Consensus statement for standard of care in spinal muscular atrophy. J Child Neurol. (2007) 22:1027–49. 10.1177/0883073807305788 17761659

[12] Grotto S Cuisset JM Marret S Drunat S Faure P Audebert-Bellanger S. Type 0 spinal muscular atrophy: further delineation of prenatal and postnatal features in 16 patients. J Neuromuscul Dis. (2016) 3:487–95. 10.3233/JND-160177 27911332

[13] Finkel RS Mcdermott MP Kaufmann P Darras BT Chung WK Sproule DM. Observational study of spinal muscular atrophy type I and implications for clinical trials. Neurology. (2014) 83:810–7. 10.1212/WNL.0000000000000741 25080519 PMC4155049

[14] Kaufmann P Mcdermott MP Darras BT Finkel RS Sproule DM Kang PB. Prospective cohort study of spinal muscular atrophy types 2 and 3. Neurology. (2012) 79:1889–97. 10.1212/WNL.0b013e318271f7e4 23077013 PMC3525313

[15] Liu Q Dreyfuss G. A novel nuclear structure containing the survival of motor neurons protein. EMBO J. (1996) 15:3555–65. 10.1002/j.1460-2075.1996.tb00725.x 8670859 PMC451956

[16] Bechade C Rostaing P Cisterni C Kalisch R La Bella V Pettmann B. Subcellular distribution of survival motor neuron (SMN) protein: possible involvement in nucleocytoplasmic and dendritic transport. Eur J Neurosci. (1999) 11:293–304. 10.1046/j.1460-9568.1999.00428.x 9987032

[17] Pagliardini S Giavazzi A Setola V Lizier C Di Luca M Debiasi S. Subcellular localization and axonal transport of the survival motor neuron (SMN) protein in the developing rat spinal cord. Hum Mol Genet. (2000) 9:47–56. 10.1093/hmg/9.1.47 10587577

[18] Fan L Simard LR. Survival motor neuron (SMN) protein: role in neurite outgrowth and neuromuscular maturation during neuronal differentiation and development. Hum Mol Genet. (2002) 11:1605–14. 10.1093/hmg/11.14.1605 12075005

[19] Singh RN Howell MD Ottesen EW Singh NN. Diverse role of survival motor neuron protein. Biochim Biophys Acta Gene Regul Mech. (2017) 1860:299–315. 10.1016/j.bbagrm.2016.12.008 28095296 PMC5325804

[20] Pellizzoni L Kataoka N Charroux B Dreyfuss G. A novel function for SMN, the spinal muscular atrophy disease gene product, in pre-mRNA splicing. Cell. (1998) 95:615–24. 10.1016/S0092-8674(00)81632-3 9845364

[21] Burghes AH Beattie CE. Spinal muscular atrophy: why do low levels of survival motor neuron protein make motor neurons sick? Nat Rev Neurosci. (2009) 10:597–609. 10.1038/nrn2670 19584893 PMC2853768

[22] Hensel N Claus P. The actin cytoskeleton in SMA and ALS: how does it contribute to motoneuron degeneration? Neuroscientist. (2018) 24:54–72. 10.1177/1073858417705059 28459188

[23] Giesemann T Rathke-Hartlieb S Rothkegel M Bartsch JW Buchmeier S Jockusch BM. A role for polyproline motifs in the spinal muscular atrophy protein SMN. Profilins bind to and colocalize with smn in nuclear gems. J Biol Chem. (1999) 274:37908–14. 10.1074/jbc.274.53.37908 10608857

[24] Sharma A Lambrechts A Hao Le T Le TT Sewry CA Ampe C. A role for complexes of survival of motor neurons (SMN) protein with gemins and profilin in neurite-like cytoplasmic extensions of cultured nerve cells. Exp Cell Res. (2005) 309:185–97. 10.1016/j.yexcr.2005.05.014 15975577

[25] Nölle A Zeug A Van Bergeijk J Tonges L Gerhard R Brinkmann H. The spinal muscular atrophy disease protein SMN is linked to the Rho-kinase pathway via profilin. Hum Mol Genet. (2011) 20:4865–78. 10.1093/hmg/ddr425 21920940

[26] Van Bergeijk J Rydel-Konecke K Grothe C Claus P. The spinal muscular atrophy gene product regulates neurite outgrowth: importance of the C terminus. FASEB J. (2007) 21:1492–502. 10.1096/fj.06-7136com 17317728

[27] Bowerman M Anderson CL Beauvais A Boyl PP Witke W Kothary R. SMN, profilin IIa and plastin 3: a link between the deregulation of actin dynamics and SMA pathogenesis. Mol Cell Neurosci. (2009) 42:66–74. 10.1016/j.mcn.2009.05.009 19497369

[28] Simic G Mladinov M Seso Simic D Jovanov Milosevic N Islam A Pajtak A. Abnormal motoneuron migration, differentiation, and axon outgrowth in spinal muscular atrophy. Acta Neuropathol. (2008) 115:313–26. 10.1007/s00401-007-0327-1 18075747

[29] Harding BN Kariya S Monani UR Chung WK Benton M Yum SW. Spectrum of neuropathophysiology in spinal muscular atrophy type I. J Neuropathol Exp Neurol. (2015) 74:15–24. 10.1097/NEN.0000000000000144 25470343 PMC4350580

[30] Moon LDF. Chromatolysis: do injured axons regenerate poorly when ribonucleases attack rough endoplasmic reticulum, ribosomes and RNA? Dev Neurobiol. (2018) 78:1011–24. 10.1002/dneu.22625 30027624 PMC6334169

[31] Martinez-Hernandez R Bernal S Also-Rallo E Alias L Barcelo MJ Hereu M. Synaptic defects in type I spinal muscular atrophy in human development. J Pathol. (2013) 229:49–61. 10.1002/path.4080 22847626

[32] Mentis GZ Blivis D Liu W Drobac E Crowder ME Kong L. Early functional impairment of sensory-motor connectivity in a mouse model of spinal muscular atrophy. Neuron. (2011) 69:453–67. 10.1016/j.neuron.2010.12.032 21315257 PMC3044334

[33] Kölbel H Hauffa BP Wudy SA Bouikidis A Della Marina A Schara U Hyperleptinemia in children with autosomal recessive spinal muscular atrophy type I-III. PLoS ONE. (2017) 12:e0173144 10.1371/journal.pone.0173144 28278160 PMC5344335

[34] Arnold AS Gueye M Guettier-Sigrist S Courdier-Fruh I Coupin G Poindron P. Reduced expression of nicotinic AChRs in myotubes from spinal muscular atrophy I patients. Lab Invest. (2004) 84:1271–8. 10.1038/labinvest.3700163 15322565

[35] Rudnik-Schoneborn S Vogelgesang S Armbrust S Graul-Neumann L Fusch C Zerres K. Digital necroses and vascular thrombosis in severe spinal muscular atrophy. Muscle Nerve. (2010) 42:144–7. 10.1002/mus.21654 20583119

[36] Araujo A Araujo M Swoboda KJ. Vascular perfusion abnormalities in infants with spinal muscular atrophy. J Pediatr. (2009) 155:292–4. 10.1016/j.jpeds.2009.01.071 19619755 PMC3250227

[37] Somers E Lees RD Hoban K Sleigh JN Zhou H Muntoni F. Vascular defects and spinal cord hypoxia in spinal muscular atrophy. Ann Neurol. (2016) 79:217–30. 10.1002/ana.24549 26506088

[38] Bowerman M Swoboda KJ Michalski JP Wang GS Reeks C Beauvais A. Glucose metabolism and pancreatic defects in spinal muscular atrophy. Ann Neurol. (2012) 72:256–68. 10.1002/ana.23582 22926856 PMC4334584

[39] Deguise MO Baranello G Mastella C Beauvais A Michaud J Leone A. Abnormal fatty acid metabolism is a core component of spinal muscular atrophy. Ann Clin Transl Neurol. (2019) 6:1519–1532. 10.1002/acn3.50855 31402618 PMC6689695

[40] Nash LA Burns JK Chardon JW Kothary R Parks RJ. Spinal muscular atrophy: more than a disease of motor neurons? Curr Mol Med. (2016) 16:779–92. 10.2174/1566524016666161128113338 27894243

[41] Tizzano EF Finkel RS. Spinal muscular atrophy: a changing phenotype beyond the clinical trials. Neuromuscul Disord. (2017) 27:883–9. 10.1016/j.nmd.2017.05.011 28757001

[42] Martinez TL Kong L Wang X Osborne MA Crowder ME Van Meerbeke JP. Survival motor neuron protein in motor neurons determines synaptic integrity in spinal muscular atrophy. J Neurosci. (2012) 32:8703–15. 10.1523/JNEUROSCI.0204-12.2012 22723710 PMC3462658

[43] Gavrilina TO Mcgovern VL Workman E Crawford TO Gogliotti RG Didonato CJ. Neuronal SMN expression corrects spinal muscular atrophy in severe SMA mice while muscle-specific SMN expression has no phenotypic effect. Hum Mol Genet. (2008) 17:1063–75. 10.1093/hmg/ddm379 18178576 PMC2835541

[44] Rindt H Feng Z Mazzasette C Glascock JJ Valdivia D Pyles N. Astrocytes influence the severity of spinal muscular atrophy. Hum Mol Genet. (2015) 24:4094–102. 10.1093/hmg/ddv148 25911676 PMC5007659

[45] Sleigh JN Gillingwater TH Talbot K. The contribution of mouse models to understanding the pathogenesis of spinal muscular atrophy. Dis Model Mech. (2011) 4:457–67. 10.1242/dmm.007245 21708901 PMC3124050

[46] Finkel RS Chiriboga CA Vajsar J Day JW Montes J De Vivo DC. Treatment of infantile-onset spinal muscular atrophy with nusinersen: a phase 2, open-label, dose-escalation study. Lancet. (2016) 388:3017–26. 10.1016/S0140-6736(16)31408-8 27939059

[47] Hoy SM. Nusinersen: first global approval. Drugs. (2017) 77:473–9. 10.1007/s40265-017-0711-7 28229309

[48] Rigo F Chun SJ Norris DA Hung G Lee S Matson J. Pharmacology of a central nervous system delivered 2'-O-methoxyethyl-modified survival of motor neuron splicing oligonucleotide in mice and nonhuman primates. J Pharmacol Exp Ther. (2014) 350:46–55. 10.1124/jpet.113.212407 24784568 PMC4056267

[49] Finkel RS Mercuri E Darras BT Connolly AM Kuntz NL Kirschner J. Nusinersen versus sham control in infantile-onset spinal muscular atrophy. N Engl J Med. (2017) 377:1723–32. 10.1056/NEJMoa1702752 29091570

[50] Luu KT Norris DA Gunawan R Henry S Geary R Wang Y. Population pharmacokinetics of nusinersen in the cerebral spinal fluid and plasma of pediatric patients with spinal muscular atrophy following intrathecal administrations. J Clin Pharmacol. (2017) 57:1031–41. 10.1002/jcph.884 28369979

[51] Mendell JR Al-Zaidy S Shell R Arnold WD Rodino-Klapac LR Prior TW. Single-dose gene-replacement therapy for spinal muscular atrophy. N Engl J Med. (2017) 377:1713–22. 10.1056/NEJMoa1706198 29091557

[52] Mercuri E Darras BT Chiriboga CA Day JW Campbell C Connolly AM. Nusinersen versus sham control in later-onset spinal muscular atrophy. N Engl J Med. (2018) 378:625–35. 10.1056/NEJMoa1710504 29443664

[53] De Vivo DC Bertini E Swoboda KJ Hwu WL Crawford TO Finkel RS. Nusinersen initiated in infants during the presymptomatic stage of spinal muscular atrophy: interim efficacy and safety results from the Phase 2 NURTURE study. Neuromuscul Disord. (2019) 29:842–56. 10.1016/j.nmd.2019.09.007 31704158 PMC7127286

[54] Hua Y Sahashi K Rigo F Hung G Horev G Bennett CF. Peripheral SMN restoration is essential for long-term rescue of a severe spinal muscular atrophy mouse model. Nature. (2011) 478:123–6. 10.1038/nature10485 21979052 PMC3191865

[55] Long KK O'shea KM Khairallah RJ Howell K Paushkin S Chen KS. Specific inhibition of myostatin activation is beneficial in mouse models of SMA therapy. Hum Mol Genet. (2019) 28:1076–89. 10.1093/hmg/ddy382 30481286 PMC6423420

[56] Faul F Erdfelder E Lang AG Buchner A. G^*^Power 3: a flexible statistical power analysis program for the social, behavioral, and biomedical sciences. Behav Res Methods. (2007) 39:175–91. 10.3758/BF03193146 17695343

[57] Parikshak NN Gandal MJ Geschwind DH. Systems biology and gene networks in neurodevelopmental and neurodegenerative disorders. Nat Rev Genet. (2015) 16:441–58. 10.1038/nrg3934 26149713 PMC4699316

[58] Stark C Breitkreutz BJ Chatr-Aryamontri A Boucher L Oughtred R Livstone MS. The BioGRID Interaction Database: 2011 update. Nucleic Acids Res. (2011) 39:D698–704. 10.1093/nar/gkq1116 21071413 PMC3013707

[59] Aranda B Achuthan P Alam-Faruque Y Armean I Bridge A Derow C. The IntAct molecular interaction database in 2010. Nucleic Acids Res. (2010) 38:D525–D531. 10.1093/nar/gkp878 19850723 PMC2808934

[60] Szklarczyk D Gable AL Lyon D Junge A Wyder S Huerta-Cepas J. STRING v11: protein-protein association networks with increased coverage, supporting functional discovery in genome-wide experimental datasets. Nucleic Acids Res. (2019) 47:D607–D613. 10.1093/nar/gky1131 30476243 PMC6323986

[61] Pathan M Keerthikumar S Ang CS Gangoda L Quek CY Williamson NA. FunRich: an open access standalone functional enrichment and interaction network analysis tool. Proteomics. (2015) 15:2597–601. 10.1002/pmic.201400515 25921073

[62] Hensel N Stockbrugger I Rademacher S Broughton N Brinkmann H Grothe C. Bilateral crosstalk of rho- and extracellular-signal-regulated-kinase (ERK) pathways is confined to an unidirectional mode in spinal muscular atrophy (SMA). Cell Signal. (2014) 26:540–8. 10.1016/j.cellsig.2013.11.027 24316236

[63] Hensel N Baskal S Walter LM Brinkmann H Gernert M Claus P. ERK and ROCK functionally interact in a signaling network that is compensationally upregulated in Spinal Muscular Atrophy. Neurobiol Dis. (2017) 108:352–61. 10.1016/j.nbd.2017.09.005 28916199

[64] Bowerman M Beauvais A Anderson CL Kothary R. Rho-kinase inactivation prolongs survival of an intermediate SMA mouse model. Hum Mol Genet. (2010) 19:1468–78. 10.1093/hmg/ddq021 20097679

[65] Bowerman M Murray LM Boyer JG Anderson CL Kothary R. Fasudil improves survival and promotes skeletal muscle development in a mouse model of spinal muscular atrophy. BMC Med. (2012) 10:24. 10.1186/1741-7015-10-24 22397316 PMC3310724

[66] Hensel N Ratzka A Brinkmann H Klimaschewski L Grothe C Claus P. Analysis of the fibroblast growth factor system reveals alterations in a mouse model of spinal muscular atrophy. PLoS ONE. (2012) 7:e31202. 10.1371/journal.pone.0031202 22348054 PMC3278439

[67] Branchu J Biondi O Chali F Collin T Leroy F Mamchaoui K. Shift from extracellular signal-regulated kinase to AKT/cAMP response element-binding protein pathway increases survival-motor-neuron expression in spinal-muscular-atrophy-like mice and patient cells. J Neurosci. (2013) 33:4280–94. 10.1523/JNEUROSCI.2728-12.2013 23467345 PMC6704952

[68] Genabai NK Ahmad S Zhang Z Jiang X Gabaldon CA Gangwani L. Genetic inhibition of JNK3 ameliorates spinal muscular atrophy. Hum Mol Genet. (2015) 24:6986–7004. 10.1093/hmg/ddv401 26423457 PMC4654054

[69] Schellino R Boido M Borsello T Vercelli A. Pharmacological c-Jun NH2-Terminal Kinase (JNK) pathway inhibition reduces severity of spinal muscular atrophy disease in mice. Front Mol Neurosci. (2018) 11:308. 10.3389/fnmol.2018.00308 30233310 PMC6131195

[70] Simon CM Dai Y Van Alstyne M Koutsioumpa C Pagiazitis JG Chalif JI. Converging mechanisms of p53 activation drive motor neuron degeneration in spinal muscular atrophy. Cell Rep. (2017) 21:3767–80. 10.1016/j.celrep.2017.12.003 29281826 PMC5747328

[71] Little D Valori CF Mutsaers CA Bennett EJ Wyles M Sharrack B. PTEN depletion decreases disease severity and modestly prolongs survival in a mouse model of spinal muscular atrophy. Mol Ther. (2015) 23:270–7. 10.1038/mt.2014.209 25369768 PMC4445616

[72] Ning K Pei L Liao M Liu B Zhang Y Jiang W. Dual neuroprotective signaling mediated by downregulating two distinct phosphatase activities of PTEN. J Neurosci. (2004) 24:4052–60. 10.1523/JNEUROSCI.5449-03.2004 15102920 PMC6729419

[73] Kuan CY Yang DD Samanta Roy DR Davis RJ Rakic P Flavell RA. The Jnk1 and Jnk2 protein kinases are required for regional specific apoptosis during early brain development. Neuron. (1999) 22:667–76. 10.1016/S0896-6273(00)80727-8 10230788

[74] Long J Cai L Li J Zhang L Yang H Wang T. JNK3 involvement in nerve cell apoptosis and neurofunctional recovery after traumatic brain injury. Neural Regen Res. (2013) 8:1491–9. 10.3969/j.issn.1673-5374.2013.16.006 25206445 PMC4107806

[75] Wang Y Luo W Reiser G. Proteinase-activated receptor-1 and−2 induce the release of chemokine GRO/CINC-1 from rat astrocytes via differential activation of JNK isoforms, evoking multiple protective pathways in brain. Biochem J. (2007) 401:65–78. 10.1042/BJ20060732 16942465 PMC1698669

[76] Li QM Tep C Yune TY Zhou XZ Uchida T Lu KP. Opposite regulation of oligodendrocyte apoptosis by JNK3 and Pin1 after spinal cord injury. J Neurosci. (2007) 27:8395–404. 10.1523/JNEUROSCI.2478-07.2007 17670986 PMC3401937

[77] Gourmaud S Paquet C Dumurgier J Pace C Bouras C Gray F. Increased levels of cerebrospinal fluid JNK3 associated with amyloid pathology: links to cognitive decline. J Psychiatry Neurosci. (2015) 40:151–61. 10.1503/jpn.140062 25455349 PMC4409432

[78] Sclip A Tozzi A Abaza A Cardinetti D Colombo I Calabresi P. c-Jun N-terminal kinase has a key role in Alzheimer disease synaptic dysfunction *in vivo*. Cell Death Dis. (2014) 5:e1019. 10.1038/cddis.2013.559 24457963 PMC4040696

[79] Pilato CM Park JH Kong L D'ydewalle C Valdivia D Chen KS Motor neuron loss in SMA is not associated with somal stress-activated JNK/c-Jun signaling. Hum Mol Genet. (2019) 28:3282–92. 10.1093/hmg/ddz150 31272106 PMC6859432

[80] Tedeschi A Di Giovanni S. The non-apoptotic role of p53 in neuronal biology: enlightening the dark side of the moon. EMBO Rep. (2009) 10:576–83. 10.1038/embor.2009.89 19424293 PMC2711843

[81] Simic G Seso-Simic D Lucassen PJ Islam A Krsnik Z Cviko A. Ultrastructural analysis and TUNEL demonstrate motor neuron apoptosis in Werdnig-Hoffmann disease. J Neuropathol Exp Neurol. (2000) 59:398–407. 10.1093/jnen/59.5.398 10888370

[82] Young PJ Day PM Zhou J Androphy EJ Morris GE Lorson CL. A direct interaction between the survival motor neuron protein and p53 and its relationship to spinal muscular atrophy. J Biol Chem. (2002) 277:2852–9. 10.1074/jbc.M108769200 11704667

[83] Chao C Herr D Chun J Xu Y. Ser18 and 23 phosphorylation is required for p53-dependent apoptosis and tumor suppression. EMBO J. (2006) 25:2615–22. 10.1038/sj.emboj.7601167 16757976 PMC1478190

[84] Hensel N Rademacher S Claus P. Chatting with the neighbors: crosstalk between Rho-kinase (ROCK) and other signaling pathways for treatment of neurological disorders. Front Neurosci. (2015) 9:198. 10.3389/fnins.2015.00198 26082680 PMC4451340

[85] Hollis ERII Jamshidi P Low K Blesch A Tuszynski MH. Induction of corticospinal regeneration by lentiviral trkB-induced Erk activation. Proc Natl Acad Sci USA. (2009) 106:7215–20. 10.1073/pnas.0810624106 19359495 PMC2678459

